# The Bern Birth Cohort (BeBiCo) to study the development of the infant intestinal microbiota in a high-resource setting in Switzerland: rationale, design, and methods

**DOI:** 10.1186/s12887-023-04198-5

**Published:** 2023-11-10

**Authors:** Luca Cecchini, Colette Barmaz, Maria José Coloma Cea, Hannah Baeschlin, Julian Etter, Stefanie Netzer, Leonie Bregy, Dmitrij Marchukov, Nerea Fernandez Trigo, Rachel Meier, Jasmin Hirschi, Jacqueline Wyss, Andrina Wick, Joelle Zingg, Sandro Christensen, Anda-Petronela Radan, Annina Etter, Martin Müller, Michael Kaess, Daniel Surbek, Bahtiyar Yilmaz, Andrew J. Macpherson, Christiane Sokollik, Benjamin Misselwitz, Stephanie C. Ganal-Vonarburg

**Affiliations:** 1 Department for BioMedical Research (DBMR), Department of Visceral Surgery and Medicine, University of Bern, Inselspital, Bern University Hospital, Freiburgstr. 18, 3010 Bern, Switzerland; 2 Department of Obstetrics and Gynaecology, Bern University Hospital, Inselspital, University of Bern, Friedbühlstrasse 19, 3010 Bern, Switzerland; 3 University Hospital of Child and Adolescent Psychiatry and Psychotherapy, University of Bern, Bolligenstrasse 111, Haus A, 3000 Bern, Switzerland; 4 Division of Pediatric Gastroenterology, Hepatology and Nutrition, Children’s Hospital, Inselspital, University of Bern, Freiburgstrasse 15, 3010 Bern, Switzerland

**Keywords:** Intestinal microbiome, Microbiota maturation, 16S sequencing, Metagenomic sequencing, Breastfeeding, Immune maturation, High resource environment, Childhood allergies, Child neuro-developmental outcomes, Mode of delivery

## Abstract

**Background:**

Microbiota composition is fundamental to human health with the intestinal microbiota undergoing critical changes within the first two years of life. The developing intestinal microbiota is shaped by maternal seeding, breast milk and its complex constituents, other nutrients, and the environment. Understanding microbiota-dependent pathologies requires a profound understanding of the early development of the healthy infant microbiota.

**Methods:**

Two hundred and fifty healthy pregnant women (≥20 weeks of gestation) from the greater Bern area will be enrolled at Bern University hospital’s maternity department. Participants will be followed as mother-baby pairs at delivery, week(s) 1, 2, 6, 10, 14, 24, 36, 48, 96, and at years 5 and 10 after birth. Clinical parameters describing infant growth and development, morbidity, and allergic conditions as well as socio-economic, nutritional, and epidemiological data will be documented. Neuro-developmental outcomes and behavior will be assessed by child behavior checklists at and beyond 2 years of age.

Maternal stool, milk, skin and vaginal swabs, infant stool, and skin swabs will be collected at enrolment and at follow-up visits. For the primary outcome, the trajectory of the infant intestinal microbiota will be characterized by 16S and metagenomic sequencing regarding composition, metabolic potential, and stability during the first 2 years of life. Secondary outcomes will assess the cellular and chemical composition of maternal milk, the impact of nutrition and environment on microbiota development, the maternal microbiome transfer at vaginal or caesarean birth and thereafter on the infant, and correlate parameters of microbiota and maternal milk on infant growth, development, health, and mental well-being.

**Discussion:**

The Bern birth cohort study will provide a detailed description and normal ranges of the trajectory of microbiota maturation in a high-resource setting. These data will be compared to data from low-resource settings such as from the Zimbabwe-College of Health-Sciences-Birth-Cohort study. Prospective bio-sampling and data collection will allow studying the association of the microbiota with common childhood conditions concerning allergies, obesity, neuro-developmental outcomes , and behaviour.

Trial registration

The trial has been registered at www.clinicaltrials.gov, Identifier: NCT04447742

## Introduction

### Background and rationale

The human intestine represents a large interface between the host and its environment, mediating the absorption of nutrients, minerals, and water as well as defense against pathogens. Intestinal functionality is critically shaped by the intestinal microbiota, comprising bacteria, viruses, fungi, and archaea that colonize the gastrointestinal tract [1, 2]. The intestinal microbiota maintains a mutually beneficial relationship with the host which together form a superorganism to which the microbiota contributes more than 99% of the combined genomic content [3].

Until recently, the complexity and diversity of the microbiota overwhelmed classical culture techniques. These limitations have now been surmounted by the extreme analytic depth and high- throughput of ‘omics` techniques (genomics, proteomics, metabolomics). The resultant data have shown the instability of the microbiota in early life, its later variability between individuals according to lifestyle, nutrition, ethnicity, and culture [4] and its association with the incidence and severity of human diseases [5, 6].

Colonization with mutualistic microbes starts principally at birth when the newborn contacts the bacteria of the birth canal or during maternal and environmental contact after caesarean section, except in pathologic conditions such as intrauterine infection with a pathogen. Although this dogma has been challenged during the last years, the scientific community at large believes that the unborn child within the uterus lives in a protected and sterile milieu [7, 8]. The early-life microbiota is unstable as incoming microbes initially populate open ecological niches, within a variable environment shaped by birth mode [9], breast- or formula feeding [10, 11], infant [12, 13] and maternal [14] diet, xenobiotics [15, 16] and protective factors [17, 18]. Subsequently, the microbiota matures, diversity of the microbiome and its metabolic activity increase gradually and reach far greater stability provided the environmental conditions are constant [19, 20].

Various factors are known to influence the infant microbiota [21, 22]: i) low gestational age leads to a dominant colonization with Gram-negative bacilli which in turn predispose infants to infections [23]. On the other hand, gestational age also determines the timing of intestinal colonization by Bifidobacteria [24]. ii) feeding crucially affects microbiota composition with higher levels of Bifidobacteria in breast-fed infants and higher levels of Bacteroides in formula-fed infants [25]. Vice versa, weaning in mice was associated with acquisition of a more adult microbiota [26, 27]. iii) C-section, for which the rate in Switzerland is 32.3% [28], leads to increased colonialization by opportunistic pathogens in the infant [29], to a higher similarity of the infant intestinal microbiota with the maternal cutaneous microbiota [30], and persisting differences in intestinal microbiota in some but not all [32] studies up to 12 months after delivery [27, 31, 33]. iv) Exposure to antibiotics in early life alters faecal microbiota development resulting in decreased Bifidobacteriaceae counts, accompanied by an increase in potentially pathogenic Enterobacteriacae. More frequent and longer antibiotic exposure will increase the extent of microbiota perturbations with a potentially detrimental impact on infant health [34].

Lactation critically influences the health and development of infants. In Switzerland, 74% of all children are fully breastfed in the first month of life. This proportion decreases to 27% by the 6th month of life, and after this period, the rate drops down to 1% [35]. Human milk contains oligosaccharides [36] which will reach and shape the infant intestinal microbiota [37], resulting in an enrichment of Bifidobacteria species which can represent 70-90% of the faecal community [38]. Besides maternal skin bacteria, Bifidobacteria were also identified in breast milk, suggesting transfer. Neither the impact of maternal bacteria transferred by breastfeeding nor variations of milk constituents on infant intestinal microbiota, immune development and well-being have been sufficiently studied [37].

Infant intestinal microbiota development also seems to impact on the development of the infant immune system. These effects of early life exposure and their consequences on immunity and allergic/autoimmune susceptibility can be modelled in experimental animals [12, 39–43]. For example, colonization with a microbiota in the first few weeks of life inhibits the accumulation of natural killer T (NKT) cells in the colonic lamina propria and decreases susceptibility to natural killer T (NKT) cell-mediated diseases observed in germ-free mice [41]. Likewise, germ-free animals harbour high serum IgE levels which could only be lowered if the mice were colonized with a complex microbiota before weaning [40]. Therefore, this early-life period has been described as a “window of opportunity” for the prevention of autoimmunity and other immunological reagulations [44, 45].

Early microbiota perturbations in infants have been associated with highly-relevant clinical conditions including autoimmune diseases such as type 1 diabetes mellitus [46], childhood atopy and asthma [47], childhood obesity [48], and intestinal conditions such as inflammatory bowel diseases [49]. The infant microbiota might also have an impact on the development of motor, social and cognitive functions [50] as well as abdominal pain.

From a global perspective, the most important set of microbiota-associated problems for human health is in child development. Childhood malnutrition is to a large degree resistant to simple caloric supplementation [37]. Poor health due to low-resources with inadequate nutrition, poor access to clean water, sanitation and hygiene is accompanied by chronic intestinal maladaptive and inflammatory alterations, summarized as environmental enteropathy (EE). EE can be summarized as a delayed and aberrant maturation of the intestinal microbiota [51, 52], ultimately resulting in dysfunction and failure of the microbiota metabolic organ [53].

In summary, there is excellent evidence that microbiota composition is fundamental to human health and that the microbiota undergoes critical changes within the first two years of life [27, 54]. We are planning a comprehensive analysis using advanced microbiota analysis techniques and small molecule composition of the infant stool samples as well as features of maternal microbiota and maternal milk. We will bring together these data with clinical characteristics and parameters for mode of birth as well as infant nutrition and hygiene to understand the development of the normal infant microbiota within the first two years of life. We hereby introduce the Bern Birth Cohort (BeBiCo) study, examining the trajectory of the microbiota in a high-resource setting. This will enable a better understanding of microbiota related pathologies in many conditions including environmental enteropathy in low-resource settings.

### Objectives

#### Aim of the study

This study aims to improve the understanding of the maturation of a healthy infant intestinal microbiota by considering composition, diversity, and metabolic activities by applying a combined approach containing shotgun metagenomic sequencing, mass spectrometry, and advanced computational approaches to identify critical microbial interactions that are key to health. The impact of factors including maternal microbiota, breast milk, and the Swiss environment (e.g., nutrition and lifestyle), which may influence the infant’s microbiota development and immune maturation will be examined and microbial data will be correlated to common childhood conditions and compared to microbiota trajectories in low-resource settings.

### Objectives

*Primary objective*: We aim for a deep understanding of the maturation of a healthy infant intestinal microbiota considering composition, diversity, and metabolic activities. We will characterize the composition, metabolic potential, and activity at various time points by advanced techniques (16S sequencing, shotgun metagenomic sequencing and mRNA sequencing) and the metabolites present by mass spectrometry. Advanced network analysis considering the combined predicted microbiota metabolic activity of all species and small molecules present can subsequently provide a comprehensive view of active metabolic pathways [18, 55–57]. Such an approach has already been applied in a previous study [58]. The trajectories of the microbiota of healthy infants in our cohort will establish standard values in a high-resource setting.

Secondary objectives of this study comprise a range of maternal and infant parameters summarized in Table 1

**Table 1:** Secondary objectives of the Bern birth cohort study

1) Understanding the impact of variations of the normal environment in Switzerland on microbiota development. To this end we will use parameters for nutrition and socioeconomic status, microbiota characteristics, and metabolomics and correlate those with parameters for infant development and immunity.
2) Understanding the transfer of the maternal microbiota to the infant during and after birth, depending on the mode of birth. Bacterial species (or operational taxonomic units, OTU) will be identified in the maternal microbiota in maternal stool, maternal skin, vaginal environment, as well as maternal milk and these parameters will be correlated to identified species/ OTU in the intestinal and skin microbiota of the infant at various points in time.
3) Understanding the impact of the microbiota on child development and health. Infant microbiota characteristics from the primary endpoint will be correlated with I. Parameters for physical development (size, weight, percentiles, head circumference, mid upper arm circumference, waist to hip ratio) II. Parameters for child neuro-development and behaviour (assessed by the child behaviour checklist) III. The onset of high body weight and obesity IV. New onset of allergies, asthma and eczema V. Onset/ occurrence of other pathologies (abdominal pain, including colic and non-specific abdominal pain, number of infectious complications, physician consultations outside regular preventive medical check-ups).
4) Understanding the impact of low resources with poor nutrition and poor hygiene in developing countries on the maturation of the intestinal microbiota. Children from the University of Zimbabwe College of health-Sciences birth cohort were followed in a similar manner as planned for the children from the Bern birth cohort study with the same acquisition of bio-samples [59]. Microbiota characteristics from endpoint 1 will be used to compare microbiota maturation in healthy Swiss infants to microbiota maturation in healthy Zimbabwean children as well as children with environmental enteropathy and stunted growth.
5) To understand the extent to which the maternal microbiota and maternal diet affect the immunomodulatory properties of breast milk and how those properties in turn influence immune maturation in the new-born, we will analyze the composition of stool and breast milk samples regarding metabolites, cellular components, cytokines, and miRNA. We will further use the material to test the impact of maternal milk *in vitro* (cell culture) and *in vivo* (animal model). Immunomodulatory mechanisms will be identified and correlating changes in the development and maturation of the microbiota of the newborn will be analyzed.

### Trial design

The Bern Birth Cohort study is a single centre prospective observational study, investigating healthy pregnant women and their new-borns in the City of Bern and its surrounding suburbs. A total number of 250 pregnant women ≥20 weeks of gestation will be enrolled from May 2020 until the targeted number is reached. Participants will be followed as mother-baby pairs at birth, within 0-3 days (including a potential intensive care unit stay), 10 days and 6, 10, 14, 24, 48 and 96 weeks of age. Additionally, there will be two visits at 5 and 10 years of age. At enrolment and each visit after birth, bio-samples will be acquired. The sampled biomaterial will be maternal stool, maternal skin swabs and vaginal swabs (at enrolment) and infant stool, infant skin swabs, maternal stool, maternal skin swabs and maternal milk (during follow-up visits). Information regarding mother and infant nutrition, infectious complications, allergies, abdominal pain, and psychological outcomes by questionnaires will be documented. In addition, data from the infants’ health booklet (used for documentation of diagnostic findings from routine check-ups by virtually all paediatricians in Switzerland) will be retained. We will measure the mid-upper arm circumference (MUAC) of mother and child as well as the infant waist-to-hip ratio.

## Methods: Participants, interventions, and outcomes

### Study Setting

The Inselspital Bern, is one of five University hospitals in Switzerland. It offers a wide range of services from basic care to highly specialized medicine. Participants will be recruited at the maternity department of the University hospital during prenatal check-ups. Bio-sample collection and filling out of the questionnaires will be executed in childbed and during visits at participants’ homes, which are located in the city of Bern and the greater Bern area. Due to the strong research cooperation between the Inselspital Bern and the University of Bern, samples will be analyzed at the Department for Biomedical Research (DBMR) affiliated to the University.

### Eligibility criteria

Pregnant women at or after week 20 of gestation living in the greater Bern area who are undergoing prenatal check-ups and planning to give birth at the study site, aged 18-45 years, are included. Participants should be able to understand and follow study procedures and understand informed consent available in German, French, or English. The proband should be in general good health (i.e., absence of major severe medical/ surgical/ psychiatric conditions requiring ongoing management). Minor well controlled conditions may be present. An inconspicuous pregnancy with absence of severe embryonal pathology should be expected in participants. However, morbidity diagnosed after birth will not be an exclusion criterion; e.g., infants with a neonatal intensive care unit stay will be followed by our study (Table 2).

**Table 2:** General inclusion and exclusion criteria of the Bern birth cohort study

**Inclusion criteria**:	**Exclusion criteria**:
a. Signed informed consent b. Ability to understand and follow study procedures and understand informed consent c. Age 18-45 years d. ≥Week 20 of pregnancy e. General good health, i.e., absence of major severe medical/ surgical/ psychiatric condition requiring ongoing management. Minor well controlled conditions may be present	
	a. Known severe embryonal pathology clearly precluding normal subsequent infant development, (minor conditions including twin/ triplet pregnancy, final pelvic position may be present) b. Participation in another clinical study interfering with study procedures.

### Recruitment

The Bern Birth Cohort study will recruit and enroll pregnant women attending prenatal care at the Maternity department of the Inselspital or of collaborating sites. As maternal informed consent also includes the infant, babies will be automatically enrolled into the study after birth and follow up visit will be conducted for mother-baby pairs. Follow up will be managed using appropriate communication modalities (e.g., cell phone, e-mail, or postal services).

### Who will take informed consent?

Eligible pregnant women will be approached by midwives working in the Maternity department of the Inselspital during scheduled pregnancy controls and, if interested, provided with the study’s patient information sheet in the language of their choice, either German, French, or English. During the subsequent clinical visit, the study will be explained by study physicians and informed consent will be obtained. No financial incentive will be provided, but participants will be compensated for study related expenses (e.g., travel). At the last time point, approximately 10 years after birth, an age-appropriate study explanation will be given to the child and provided he/she consents, information and bio-samples will be collected.

### Additional consent provisions for collection and use of participant data and biological specimens

Additional consent will be obtained for the use of participants’ data and biological specimens in potential ancillary studies, following Swiss regulations. In addition, consent to collect and analyze participant’s genetic information is gained as human genetic information will inevitably be gathered when performing shotgun metagenomic sequencing aiming to decipher the microbiome.

### Interventions

#### Explanation for the choice of comparators

Not applicable, non-interventional study

#### Intervention description

Not applicable, non-interventional study

#### Criteria for discontinuing or modifying allocated interventions

Not applicable, non-interventional study

#### Strategies to improve adherence to interventions

Not applicable, non-interventional study

#### Relevant concomitant care permitted or prohibited during the trial

Not applicable, non-interventional study

#### Provisions for post-trial care

Not applicable, non-interventional study

### Outcomes

#### Primary outcome

Infant intestinal taxonomy profile and its dynamics over time (see indicated time points).

Secondary outcome measurements

- 1.Association of microbiota profile to maternal and infant parameters including mode of birth, nutrition, breastfeeding, and socioeconomic status.
- 2.Similarity between the infant and maternal microbiota in stool, skin, (maternal) milk and (maternal) vagina. Metagenomic sequencing will identify bacterial strains which can be followed in maternal and infant bio-samples to demonstrate microbiota seeding.
- 3.Correlating infant microbiota parameters to:
  -Parameters for physical development (size, weight, percentiles, head circumference, upper arm circumference, waist-to-hip ratio)
  -Parameters for child neuro-developmental outcomes and behavior (assessed by the child behaviour checklist CBCL)60,61
  -Onset of high body weight and obesity
  -New onset of allergies, asthma and eczema. Onset/ occurrence of other pathologies (abdominal pain including colic and non-specific abdominal pain, number of infectious complications, physician consultations outside regular preventive medical check-ups).
- 4.Comparing the trajectory of microbiota development in the Bern area as a high-resource setting with a low-resource setting in sub-Saharan Africa (i.e. samples from the University of Zimbabwe College of Health Sciences (UZ-CHS) birth cohort)59
- 5.Characterization of maternal stool taxonomy profile and extend of similarity to maternal milk taxonomy.
- 6.Correlating breast milk composition (cellular content by flow cytometry and single-cell RNA-Sequencing, chemical composition by mass spectrometry, cytokine measurements, microbiota) to maternal nutrition and to infant microbiota and health.
- 7.Analyzing immunomodulatory properties in breast milk by the use of human and murine intestinal organoid cultures and in vivo animal models.

### Participant timeline

#### Sample size

The primary outcome of our study, “trajectory of microbiota development”, is exploratory and no power analysis is possible. However, we performed a power analysis for several secondary outcomes:

- The relative abundance of Lactobacilli in the infant intestine during the first month of life has been estimated to be 0.01 after C-section and 0.07 with a standard deviation of 0.14 after vaginal delivery [62]. The fraction of C-section in our cohort is 25% (unpublished observations). According to our power analysis [63], a sample size of 228 participants would be required to detect this difference with a power of 0.8 and a p-value of 0.05.
- In a previous study [64], a Shannon diversity index of the intestinal microbiota at the end of the first year of life was found to be 1.34 with asthma at age 7 and 1.6 in healthy children with an estimated standard deviation of the population of 0.35. The prevalence of asthma in children with asthma in Switzerland is estimated to be approximately 12% [65]. According to our power analysis, a sample size of 135 would be required to detect this difference with a power of 0.8.

These examples illustrate that our sample size of 250 is sufficient to detect a number of previously demonstrated associations of the microbiota with common clinical conditions.

For practical reasons, a sample size of 250 mother-baby pairs was defined, also according to our estimated capacities for recruitment, follow-up, sample collection, and laboratory analyses. Even with a number of 250 mother-baby pairs, we do not assume sufficient power for all outcomes. With a prevalence of 20% [66] for atopic dermatitis or 15.9% [67] for combined overweight and obesity in Swiss infants, our study may be powered sufficiently. On the other hand the estimated rate of autism spectrum disorder is expected to be 0.6 – 0.8% [68], for which our study might not find statistical significant findings. However, any preliminary results could inform future larger follow-up studies.

### Assignment of interventions: allocation

#### Sequence generation

Not applicable, non-interventional study

#### Concealment mechanism

Not applicable, non-interventional study

#### Implementation

Not applicable, non-interventional study

### Assignment of interventions: Blinding

#### Who will be blinded

Not applicable, non-interventional study

#### Procedure for unblinding if needed

Not applicable, non-interventional study

### Data collection and management

#### Plans for assessment and collection of outcomes

At enrolment, skin swabs and vaginal swabs will be collected by the maternity clinic’s midwives during routine pregnancy check-ups. Vaginal samples will be temporarily stored at room temperature. Skin swabs will be stored at room temperature for up to three hours or at -18°C until their transfer into -80°C biobank at DBMR.

A sampling kit will be provided to the participants for maternal stool collection at home. Stool samples are stored in the participant’s home freezer (typically -18°C in Switzerland), before sending of samples to DBMR via postal service and subsequent storage in the -80°C biobank. Participants are encouraged to send samples on Mondays to Thursdays to avoid shipment of samples over the weekend.

Mothers will be contacted via phone or text message by the study team and an appointment will be made within the specified time frame (Table 3). Breast milk, maternal skin swabs, infant stool, and infant skin swabs are all collected at the participant’s home. For the acquisition of 1-20 ml milk samples manual expression of the breast will be performed by the mother or a milk pump will be used. The breast will not be cleaned before sampling in order to preserve representative conditions of breastfeeding. Infant stool samples will be acquired within three hours after defecation from infant diapers; alternatively, if this timeframe cannot be met, a sampling kit for fresh stool collection will be provided to the mothers, which will be retrieved at the next research visit. Skin swabs will be done in the inguinal region. Biological specimens are immediately stored on dry ice in mobile boxes before transfer to the -80°C biobank. Milk will be aliquoted before freezing on dry ice and 5 ml will be kept unfrozen and transported on ice to the research institute for flow cytometric analysis. The transfer of the community acquired samples into the -80°C Biobank lasts on average 30 minutes and at maximum 1.5 hours. In case of collection of stool and skin swab samples in the absence of the study team, participants will store the samples in their personal -18°C freezer until hand over to the study team.

**Table 3:** Study procedures timeline

**Timepoints ±tolerance interval** **(birth=0)**	**Enrolment**	**Research visit (days)**		**Research visit (weeks)** **(years)**						**Research visit**		
		**0-3** **±3**	**10** **±5**	**6** **±2**	**10** **±4**	**14** **±4**	**24** **±6**	**36** **±8**	**48** **±8**	**96** **±12**	**5** **±1**	**10** **±1**
**Information about the study**	**+**											
**Check inclusion/** **exclusion criteria**	**+**											
**Signing of written informed consent**	**+**											
**Medical history**	**+**											
**Baseline questionnaire**	**+**											
**3-days weighed food record of the mother (optional)**	**+**			**+**		**+**	**+**					
**Food frequency questionnaire of the mother**	**+**			**+**		**+**	**+**					
**3-days weighed food record of the infant (optional)**									**+**	**+**	**+**	**+**
**Food frequency questionnaire of the infant**									**+**	**+**	**+**	**+**
**Follow-up questionnaire**			**+**	**+**	**+**	**+**	**+**	**+**	**+**	**+**	**+**	**+**
**Anthropometric measures**			**+**	**+**	**+**	**+**	**+**	**+**	**+**	**+**	**+**	**+**
**Maternal stool samples**	**+**		**+**				**+**			**+**	**+**	**+**
**Maternal skin swab**	**+**		**+**				**+**			**+**	**+**	**+**
**Maternal vaginal samples**	**+**											
**Maternal milk samples, if nursing**			**+**	**+**	**+**	**+**	**+**	**+**	**+**	**+**		
**Infant stool samples**		**+**	**+**	**+**	**+**	**+**	**+**	**+**	**+**	**+**	**+**	**+**
**Infant skin swabs**		**+**	**+**	**+**	**+**	**+**	**+**	**+**	**+**	**+**	**+**	**+**
**Child behaviour check list (CBCL)**										**+**	**+**	**+**

Microbial populations and its metabolic activities within infant stool will be analyzed using several approaches: i) 16S rRNA amplicon sequencing, ii) shotgun metagenomic sequencing, iii) bacterial mRNA sequencing and iv) mass spectrometry.

For *16S rRNA amplicon sequencing,* paired-end MiSeq Illumina reads (2 × 300 bp) will be analyzed using the QIIME2 2022.2 pipeline as described [69] using custom scripts for analysis on the UBELIX high-performance cluster at the University of Bern. The software package Divisive Amplicon Denoising Algorithm 2 (DADA2) [70] will be used to infer true biological sequences from reads.

A deeper resolution will be then obtained using *Shotgun metagenomic sequencing data* which will be generated within NovaSeq Illumina reads (2 × 150 bp) in paired-end mode aiming to cover 50 million reads per sample from the sequencer. Data will be analyzed for taxonomy profile using Kraken2 [71] with a customized database that enables profiling of bacteria, archaea, prokaryotes and viruses. *Bacterial mRNA sequencing*will be done as previously described [72].

*Mass spectrometry*will be used to assess the metabolome of the intestinal content as done in previous studies [56, 57, 72]. Briefly, metabolites from the collected intestinal content will be extracted by homogenization with 80°C hot Millipore water in a tissue homogenizer. Samples are then incubated in a thermomixer, vortexed and centrifugated. The supernatant will be collected and sealed in plates before relative metabolite ion abundance will be quantified by a mass spectrometer. Ions will be annotated based on an accurate mass comparison against 9,261 unique metabolites present in the Human Metabolome Database. For targeted metabolomic analyses, more specific protocols will be applied. For the secondary outcomes some analyses will be performed in parallel on infant and maternal skin swabs, maternal milk, maternal vaginal swabs, and maternal stool. Analysis methods may still change during the course of the study. At any of the time we will use the most up-to-date and appropriate methods.

*Flow cytometry and fluorescence-activated cell sorting (FACS)*will be performed in fresh breast milk samples to assess cellular (leukocytes, stem cells, epithelial cells etc.) composition and analyzed with FlowJo software. Multiplex enzyme-linked immunosorbent assay (ELISA and/or Mesoscale Scale Discovery) will be used to determine the cytokine and antibody content. In addition, we will employ proteomic and untargeted as well as targeted mass spectrometry approaches to determine the metabolome of breast milk similar to previous work with other biosamples [56, 57, 72].

*Biological activity screening using small intestinal organoids* of murine or human origin [73–75] will be incubated with filtered and diluted breast milk aliquots followed by analyses of the intestinal organoids (histology, RT-qPCR) to assess bioactive compounds in breast milk, e.g. arylhydrocarbon receptor ligands [76] and cytokines.

### Mother and infant anthropometric, sociodemographic, health, and neuro-developmental assessment

Infant health information such as infant growth characterized by weight size and head circumference, or altered immune maturation revealed by occurrence of asthma, atopy or allergies and potential occurrence of morbidity will be acquired using questionnaires at enrolment and at every follow-up visit. Infant neuro-developmental outcomes will be assessed with the child behaviour checklist (CBCL), a widely used questionnaire to support assessment of behavioural and emotional problems [60, 61]. Maternal and infant nutrition will be assessed by the combinatory approach of using both validated food frequency questionnaires and classical 1-3-days weighted food reports. Hygiene, socioeconomic status, and clinical history of mother and infant will be assessed in questionnaires as well. Maternal anthropometric data will be measured before, during, and after pregnancy. At enrolment, complementary data from the mother’s patient dossiers will be documented. A comprehensive summary of acquired information is provided in Table 4.

**Table 4:** Maternal and infant data collection time points

**Timepoints ±tolerance interval** **(birth=0)**	**Enrolment**	**Research visit (days)**		**Research visit (weeks)** **visit (years)**						**Research**		
		**0-3** **±3**	**10** **±5**	**6** **±2**	**10** **±4**	**14** **±4**	**24** **±6**	**36** **±8**	**48** **±8**	**96** **±12**	**5** **±1**	**10** **±1**
**A. Maternal data collection**												
1. Smoking	**+**											
2. Diet and occasions of modification	**+**											
3. Physical activity	**+**											
4. Number of persons in household	**+**											
5. Presence of domestic animals	**+**											
6. Educational level	**+**											
7. Ethnicity and migration history	**+**											
8. Size and weight (before, during and after pregnancy)	**+**		**+**									
9. Presence of recurring abdominal pain	**+**											
10. Presence of pregnancy complication (e.g. preeclampsia/eclampsia, gestational diabetes)	**+**											
11. History of previous births	**+**											
12. Medical history	**+**											
13. Current medication	**+**											
**B. Infant data collection**												
1. Twin or triplet pregnancy			**+**									
2. Sex	**+**		(+)									
3. Birth mode			**+**									
4. Birth complications			**+**									
5. Nutrition and reason for modifications			**+**	**+**	**+**	**+**	**+**	**+**	**+**	**+**	**+**	**+**
6. Presence of colic			**+**	**+**	**+**	**+**	**+**	**+**	**+**	**+**	**+**	**+**
7. Growth parameter (Weight, Size, Head circumference)			**+**	**+**	**+**	**+**	**+**	**+**	**+**	**+**	**+**	**+**
8. Body proportion (Mid-upper arm, abdominal and waist circumference)			**+**	**+**	**+**	**+**	**+**	**+**	**+**	**+**	**+**	**+**
9. Occurrence end extent of third-party care			**+**	**+**	**+**	**+**	**+**	**+**	**+**	**+**	**+**	**+**
10. Presence of developmental disorders			**+**	**+**	**+**	**+**	**+**	**+**	**+**	**+**	**+**	**+**
11. Allergies			**+**	**+**	**+**	**+**	**+**	**+**	**+**	**+**	**+**	**+**
12. Episodes of fever			**+**	**+**	**+**	**+**	**+**	**+**	**+**	**+**	**+**	**+**
13. Presence of any disease (e.g. bronchitis or gastroenteritis)			**+**	**+**	**+**	**+**	**+**	**+**	**+**	**+**	**+**	**+**
14. Emergency consultations			**+**	**+**	**+**	**+**	**+**	**+**	**+**	**+**	**+**	**+**
15. Antibiotic therapy			**+**	**+**	**+**	**+**	**+**	**+**	**+**	**+**	**+**	**+**
16. Vaccines			**+**	**+**	**+**	**+**	**+**	**+**	**+**	**+**	**+**	**+**
17. Medication			**+**	**+**	**+**	**+**	**+**	**+**	**+**	**+**	**+**	**+**
18. Physical activity				**+**	**+**	**+**	**+**	**+**	**+**	**+**	**+**	**+**

### Plans to promote participant retention and complete follow-up

For convenient follow-up, home visits will be offered to mothers to lower the workload and time investments for the mothers to a minimum. Mothers will be contacted early to find a suitable date for appointment. For all follow-up time points, a time window is defined within an interval, providing flexibility for the study team and participants. At the visits, the study team will continuously provide information about postnatal microbiome development to interested participants. Enrolled mothers will obtain an update on the progression of the study every two years to promote retention to the study until the 10-year follow up time point. We aim for a drop-out rate below 15% over 10 years.

### Data management

Study data are recorded in the worldwide used research electronic data capture application REDCap^TM^. This study database is run by the Clinical Trial Unit (CTU) of the University of Bern, Switzerland. Data will be physically stored on daily backed-up servers of the University of Bern, located in Bern, Switzerland. Source data will be collected during screening and follow-up, coded, and entered into the electronic database (electronic case report form, eCRF). Where applicable, format, range, and mandatory fields where content must be entered will be defined. For analysis, data will be exported as comma-separated values (csv) files.

### Retention and destruction of study data and biological material

The data and samples collected during this study will be stored for at least 10 years after the end of the study. It is possible that the data and samples will be used for subsequent projects or be sent to a different database/biobank in Switzerland or abroad for further studies not yet defined. At enrolment, participants will be asked for permission for further use of data and samples.

After termination of the study, samples will be autoclaved and put to the waste which will subsequently be burned. These procedures will be documented in our study documentation.

### Confidentiality

Identifying participant data will be handled with uttermost discretion and are only accessible to authorized personnel who require the data to fulfil their duties within the scope of the research project. On the CRFs and other project specific documents, participants are only identified by a unique participant number. The anonymity of the participants will be guaranteed when presenting the data at scientific meetings or publishing them in scientific journals. Disclosure of individual clinical information to third parties is prohibited.

### Plans for collection, laboratory evaluation and storage of biological specimens for genetic or molecular analysis in this trial/future use

Biological material in this project is not identified by participant name but by a unique study ID. Biological material is stored at -80°C in a restricted area only accessible to authorized personnel. All freezers have temperature control implemented (temperature is continuously monitored) and are alarm protected.

### Statistical methods

#### Statistical methods for primary and secondary outcomes

Observed effects and interactions will be statistically substantiated by parametric and non- parametric tests and/ or multivariate analyses. A *p*-value <0.05 will be considered significant. For microbiota data, false discovery rate correction with the Benjamini-Hochberg method will be used and a q-value <0.05 will be considered significant.

For an overview of sample composition, α- and β-diversity assessment and a microbiota clustering analysis based on a Bray-Curtis dissimilarity principal coordinate analysis (PCoA) or a principal component analysis (PCA), will be performed. A pipeline for detailed assessment of 16S rRNA amplicon sequence data (Quantitative Insights Into Microbial Ecology QIIME2 and several R packages including phyloseq, ggplot2, shape, ape, vegan, and MaAslin2) for assessment of diversity and PCoA will be used as done previously [77–79].

To analyse the complex composition of bacterial communities, the extraction of the representative sequences using the “feature-table” and their classification by taxon using the “feature-classifier” will be performed. The SILVA [80] (version 132) database will be used to identify the taxonomy of each representative sequence. The taxonomy.gza, rep-seqs.qza and rooted-tree.qza files generated in QIIME2 will be called out in *phyloseq* pipeline in R for downstream analysis [81, 82].

To find associations between clinical metadata and microbial community parameters, the multivariate analysis by linear models (MaAsLin2) will be used [83]. In addition, all-to-all hierarchical clustering analysis (HAIIA) (https://huttenhower.sph.harvard.edu/halla) will be applied for microbiota data and clinical metadata as well as milk composition and 16DS data to identify the critical parameters that influence microbiota profile [78].

For functional profiling of the microbiota communities from shotgun metagenomic sequencing will be performed. Data will be analyzed with the HUMAnN pipeline to check for the presence and abundance of microbial pathways. Information about metabolic pathways in the microbiota helps us understand the relationship between microbiota composition and possible substrates and metabolites in the assessed samples.

Microbiota analyses methods are quickly evolving, and we will continuously adapt and refine our analysis pipeline.

#### Interim analyses

No criteria for a premature stop are defined, due to the explorative nature of our study. An interim analysis will be performed for the primary and selected secondary outcomes, when a sample size of fifty mother-baby pairs is reached. We plan to publish an interim analysis within the next two-three years.

#### Methods for additional analyses (e.g., subgroup analyses)

Not applicable

#### Methods in analysis to handle protocol non-adherence and any statistical methods to handle missing data

The nature of this exploratory study allows for some tolerance regarding missing data. Patients with a limited number of missing samples will not be replaced if microbiota assessment is not compromised (no threshold provided). Patients with an insufficient number of bio-samples and dropouts within the first 2 years will be replaced by newly recruited individuals, until the final number of 250 individuals will be reached.

#### Plans to give access to the full protocol, participant level-data and statistical code

The full study protocol is accessible under www.clinicaltrials.gov under the number NCT04447742. Participant level-data and statistical code will be published together with the results of our analyses. However, some restrictions regarding sharing of individual participant data apply according to Swiss law.

### Oversight and monitoring

#### Composition of the coordinating centre and trial steering committee

The sponsor will also act as coordinating principal investigator (CPI) and take general responsibility for the study, coordinate modifications in study design, data collection, trial management, safety reporting, analysis and interpretation, manuscript and clinical report writing, and spreading of results.

The CPI will build up and lead a trial management group (TMG), which will include, representatives from the co-involved clinics and coinvestigators. Daily tasks and activities will be managed by the coinvestigators and dedicated study personnel who are accountable to the CPI.

#### Composition of the data monitoring committee, its role and reporting structure

All involved investigators will allow monitoring, audits, or regulatory inspections on behalf of the sponsor or the local ethics committee. Monitoring will be performed by monitoring designees according and performed continuously throughout the study starting at involvement of the first participant. The monitoring will include trial master file review, review of completeness of informed consent forms, source data verification and serious adverse event review.

#### Adverse event reporting and harms

If a serious event occurs, the study members will contact the CPI within 24 hours via telephone. The research project will be interrupted and the Ethics Committee notified on the circumstances via Business Administration System for Ethics Committees (BASEC) within 7 days, according to the Swiss Human Right Ordinance (HRO) Art. 211 [84].

#### Frequency and plans for auditing trial conduct

The study will be performed in line with the principles of the declaration of Helsinki and the International Conference on Harmonisation Tripartite Guideline for Good Clinical Practice (ICH GCP). However, besides monitoring, no formal auditing is planned.

#### Plans for communicating important protocol amendments to relevant parties (e.g. trial participants, ethical committees)

For important protocol changes, amendments of the study protocol will be proposed to the local Ethics Committee for approval. The amendment will be drafted by the CPI after consultation with the trial management team. Participants will be informed at the next visit and, if applicable will undersign a new informed consent.

#### Dissemination plans

A television broadcast in a scientific TV program presenting the concept of the study has already been realized. The content of the program is still available online[85]. Analyzed datasets will be published in peer-reviewed scientific journals and presented at international scientific conferences. Outreach to the lay public via our website or press releases are also planned. As mentioned above enrolled mothers will obtain an update on the progression of the study every two years until the 10-year follow up time point.

## Discussion

The Bern Birth Cohort study will include 250 healthy mother-baby pairs in the Swiss city of Bern and its suburb area. We will perform deep clinical phenotyping regarding maternal health and nutrition, infant growth, development, neuro-developmental outcomes, and morbidities. Bio-samples will cover maternal milk samples, maternal and infant stool samples, maternal vaginal samples as well as skin swabs. For the appropriate specimen, microbial, chemical and/or cellular composition will be analyzed by state-of-the-art techniques. Correlating characteristics of the infant microbiome with clinical and extensive data can reveal the impact of the microbiota on the health of the new-born. This may be relevant for three common conditions: obesity, allergic conditions and mental disorders.

In earlier studies, it has been shown that the microbiota increases fat storage in adipocytes and microbiota composition has been proposed to be a relevant environmental factor for obesity [86]. Furthermore, obesity coincides with a decreased intestinal microbiome diversity [87]. There are two dominant phyla of commensal bacteria in the human intestine, the Bacteroidetes and the Firmicutes. The ratio of Bacteroidetes to Firmicutes is decreased in obese individuals [88]. Non-digestible carbohydrates are fermented by the gut microbiota to short-chain fatty acids (SCFAs). SCFAs, when produced in an increased quantity, lead to an excessive calory supply and promotion of obesity [89]. Most previous studies have analyzed microbiome composition in obese individuals and compared it to lean controls [90, 91]. Longitudinal changes in the microbiome composition preceding obesity have been investigated before [92, 93], but our study might yield important additional insights regarding mechanistic aspects as well as the time course of changes in microbiota and body weight.

Infant intestinal microbiota development seems to influence the formation and maturation of the infant immune system. Observations from independent birth cohorts showed that the absence of specific gut bacteria (e.g. *Achnospira, Veillonella, Faecalibacterium*, and *Rothia*) increased risks of atopy, recurrent wheeze, or asthma development in childhood, and suggested an underlying T cell dysfunction [47, 94, 95]. One-month-old infants with high risk for development of atopy or asthma later in childhood characteristically showed depletion of dihomo-γ- linoleate, a precursor for anti-inflammatory prostaglandins, and docosapentanoic acid, an anti-inflammatory ω-3 polyunsaturated fatty acid [95]. While a low gut microbiome diversity does not necessarily favour the onset of asthma, a surplus of particular strains of bacteria including Bacteroidaceae, Clostridiaceae and Enterobacteriaceae facing a reduced proportion of Lactobacillaceae, Lachnospiraceae, Veillonellaeceae and Verrucomicrobiaceae were associated with increased prevalence of asthmatic disorders [96]. Consequently the microbial imbalance entails a reduced synthesis of SCFA (butyrate, acetate, propionate), which are important for immune signaling pathways [97]. In our study the analysis of the trajectory of the gut microbiota maturation and its influencing environmental factors (breastfeeding, maternal and infant nutrition, lifestyle) may confirm specific correlations between clinical and microbiota data [10, 11, 13, 98, 99] but also identify new associations. A potential extension of our study could be the isolation of beneficial (probiotic) strains which could support the maturation of the microbiota for various health benefits.

Several animal studies hint that altered assembly of the infant microbiome can influence neurodevelopment and may lead to atypical motor, social–emotional and cognitive development. The infant microbiota maturation coincides with the critical period of early brain development. Interestingly, in a germ-free (GF) mouse model, expression of synaptogenesis related proteins is increased; however, hypermyelination was normalised after colonizing GF mice with a conventional microbiota [100]. An accelerated brain growth in infancy is associated with neurological impairments (e.g. delays in motor, language, and cognitive functions) [50]. A consequence of accelerated brain growth can include atypical brain connectivity patterns, which are associated to neuro-development disorders including autism spectrum disorders (ASD) [101, 102]. The resident immune cells of the central nervous system not only play a role in inflammation but also in the forming process of neural circuits in the developing brain [103]. Once again the activity and function of the microglia is influenced by the hosts microbiome [104]. Importantly, many ASD patients suffer from gastrointestinal problems, including diarrhea, obstipation and abdominal pain [105, 106]. By using the CBCL, infants can be stratified according to behavioural patterns including aggressiveness, impulsivity, hyperactivity anxiety. CBCL can also inform physicians about a potential risk for mental health conditions such as ASD, depression and attention deficit hyperactivity disorder (ADHD). CBCL information will be associated with relevant parameters of the gut microbiota.

The role of the local environment (low- vs. high-resource setting) on the developing intestinal microbiome will be illustrated by comparing this cohort to a very similar study, the University of Zimbabwe College of health Sciences cohort [59]. The Zimbabwean cohort started in 2016 and primarily examines the impact of HIV and early life antiretroviral therapy (ART) exposures on different paediatric outcomes and has now recruited a total of 1200 mother-baby pairs. The study shares a very similar study design with identical follow-up timepoints, similar information gathering concerning clinical, socio-economic, nutritional, and environmental data, and similar bio-sampling methods for maternal stool, milk, and infant stool allowing to compare the microbiota development in highly different environments.

Strengths of our study include a homogenous study population in a high-resource setting, longitudinal analysis starting before birth, systematic extensive bio-sampling, and collection of a comprehensive dataset on infant and maternal health and lifestyle. Limitations include lack of acquisition of infant or maternal blood or blood cord samples. Moreover, no screening of the mothers for depression, anxiety, or stress, and no formal assessment of child development is performed. Biosampling for microbiota analyses will invariably assess only a fraction of the respective compartment, and e.g., the inguinal region might not be representative of the skin microbiome and confounded by diaper wearing. Further, our sample size might have limited power to detect associations between microbiota parameters and infant health, except for very common conditions such as obesity, and conditions related to allergies. An extension of the cohort size will be possible in the future.

In conclusion, the BeBiCo study will provide a comprehensive and detailed picture of microbiota maturation in the child from birth onwards in a high-resource setting and may support our understanding of microbiota-related pathologies in childhood and later life.

### Trial status

The current protocol version is version 7.0 dated 20^th^ of May 2022. Recruitment was launched on the 1^st^ of May 2020 and the first participant was included on the 18^th^ of May 2020. We expect the completion of recruitment by December 2025.

## Role of sponsor

The sponsor holds a dual function as coordinating principal investigator (CPI) and sponsor. The sponsor will design and manage the study in cooperation with the study team.

## Data Availability

The anonymized datasets and statistical code used and/or analyzed in the current study will be published together with results. Additional information will be available from the corresponding author on reasonable request. There are no contractual agreements limiting data access but some restrictions to sharing of individual participant data according to Swiss law exist.

## Abbreviations

ADHD: Attention deficiency hyperactivity disorder
ART: Antiretroviral therapy
ASD: Autism spectrum disorders
BASEC: Business Administration System for Ethics Committees
BeBiCo: Bern birth cohorts
BMI: Body mass index
CPI: Coordinating principal investigator
CTU: Clinical trials unit
DBMR: Department of biomedical research
EE: Environmental enteropathy
eCRF: Electronic case report form
ELISA: Enzyme-linked immunosorbent assay
FACS: Fluorescence-activated cell sorting
GCP: Good clinical practice
GF: Germ-free
HAIIA: Hierarchical clustering analysis
HRO: Human rights ordinance
MUAC: mid-upper arm circumference
OTU: Operational taxonomic unit
PCA: Principal component analysis
PCoA: Principal coordinate analysis
QIIME: Quantitative insights into microbial ecology
SCFA: Short-chain fatty acid
SSL: Secure Sockets Layer
TMG: Trial management group
